# Incremental diagnostic value of [^18^F]tetrafluoroborate PET-CT compared to [^131^I]iodine scintigraphy in recurrent differentiated thyroid cancer

**DOI:** 10.1007/s00259-020-04727-9

**Published:** 2020-04-04

**Authors:** Matthias Dittmann, José Manuel Gonzalez Carvalho, Kambiz Rahbar, Michael Schäfers, Michael Claesener, Burkhard Riemann, Robert Seifert

**Affiliations:** 1 Department of Nuclear Medicine, University Hospital Münster, Albert-Schweitzer-Campus 1, 48149 Münster, Germany; 2 European Institute for Molecular Imaging (EIMI), University of Münster, Münster, Germany; 3 Cells in Motion Interfaculty Centre (CiM), University of Münster, Münster, Germany

**Keywords:** Thyroid cancer, Tetrafluoroborate, Iodine, PET-CT, SPECT, WBS

## Abstract

**Introduction:**

Efficient therapy of recurrent differentiated thyroid cancer (DTC) is dependent on precise molecular imaging techniques targeting the human sodium iodide symporter (hNIS), which is a marker both of thyroid and DTC cells. Various iodine isotopes have been utilized for detecting DTC; however, these come with unfavorable radiation exposure and image quality ([^131^I]iodine) or limited availability ([^124^I]iodine). In contrast, [^18^F]tetrafluoroborate (TFB) is a novel radiolabeled PET substrate of hNIS, results in PET images with high-quality and low radiation doses, and should therefore be suited for imaging of DTC. The aim of the present study was to compare the diagnostic performance of [^18^F]TFB-PET to the clinical reference standard [^131^I]iodine scintigraphy in patients with recurrent DTC.

**Methods:**

Twenty-five patients with recurrent DTC were included in this retrospective analysis. All patients underwent [^18^F]TFB-PET combined with either CT or MRI due to newly discovered elevated TG levels, antiTG levels, sonographically suspicious cervical lymph nodes, or combinations of these findings. Correlative [^131^I]iodine whole-body scintigraphy (dxWBS) including SPECT-CT was present for all patients; correlative [^18^F]FDG-PET-CT was present for 21 patients. Histological verification of [^18^F]TFB positive findings was available in 4 patients.

**Results:**

[^18^F]TFB-PET detected local recurrence or metastases of DTC in significantly more patients than conventional [^131^I]iodine dxWBS and SPECT-CT (13/25 = 52% vs. 3/25 = 12%, *p* = 0.002). The diagnosis of 6 patients with cervical lymph node metastases that showed mildly increased FDG metabolism but negative [^131^I]iodine scintigraphy was changed: [^18^F]TFB-PET revealed hNIS expression in the metastases, which were therefore reclassified as only partly de-differentiated (histological confirmation present in two patients). Highest sensitivity for detecting recurrent DTC had the combination of [^18^F]TFB-PET-CT/MRI with [^18^F]FDG-PET-CT (64%).

**Conclusion:**

In the present cohort, [^18^F]TFB-PET shows higher sensitivity and accuracy than [^131^I]iodine WBS and SPECT-CT in detecting recurrent DTC. The combination of [^18^F]TFB-PET with [^18^F]FDG-PET-CT seems a reasonable strategy to characterize DTC tumor manifestations with respect to their differentiation and thereby also individually plan and monitor treatment. Future prospective studies evaluating the potential of [^18^F]TFB-PET in recurrent DTC are warranted.

## Introduction

The diagnostic workup of patients with recurrent differentiated thyroid cancer (DTC) requires molecular imaging strategies targeting the human sodium iodide symporter (hNIS), which is a key molecular marker of thyroid and DTC cells [1]. The radiopharmaceuticals [^124^I]iodide, [^123^I]iodide, and [^131^I]iodide are substrates of hNIS and thus are predominately used for molecular imaging of recurrent DTC [2]. However, positron emitting [^124^I]iodine is of subordinate diagnostic significance, as it is only available at few sites and delivers image acquisitions with a low signal to noise ratio [2]. Therefore, diagnostic [^131^I]iodine or [^123^I]iodine whole-body scintigraphy (dxWBS) and single photon emission tomography (SPECT-CT) are the current clinical reference standards for the diagnostic workup of DTC patients. Yet both iodine dxWBS and SPECT-CT achieve only unsatisfactory image quality, especially when compared to positron emission tomography (PET) imaging approaches [3–6]. Moreover, [^131^I]iodine causes significant radiation exposure, and the administration might require an in-patient stay due to radiation protection legislation [7].

The detection of local recurrence or metastases of DTC by iodine imaging is of great relevance for the planning of localized therapies, like surgery or radiation therapy [2]. Moreover, negative iodine imaging despite elevated tumor marker levels is a prerequisite for diagnosing the “Thyroglobulin Elevated and Negative Iodine Scintigraphy” (TENIS) syndrome, which requires fundamentally different diagnostic and therapy concepts [8–10]. Therefore, a failure in detecting hNIS-expressing local recurrences or metastases of DTCs might result both in a missed opportunity for localized therapy and an erroneously assumed TENIS syndrome.

Recently, [^18^F]fluoride labeled tetrafluoroborate (TFB) has been employed in the primary staging of patients with DTC [11]. [^18^F]TFB is likewise a substrate of hNIS and therefore an analogue to [^124^I]iodide or [^131^I]iodide [12]. Yet in contrast to iodine nuclides, [^18^F]TFB can easily be synthesized by cyclotrons equipped with PET radiochemistry and offers a favorable half-life, dosage and biodistribution, as well as superior PET image quality [13–15]. However, it remains unclear, if [^18^F]TFB-PET-CT is superior to conventional [^131^I]iodine dxWBS/SPECT-CT in post-radioiodine ablation (RAI) patients suffering from recurrent DTC.

Therefore, the aim of the present study was to evaluate the potential of [^18^F]TFB-PET in patients with newly discovered recurrent DTC. To this end, the clinical reference standard [^131^I]iodine dxWBS/SPECT-CT was compared on an individual basis to [^18^F]TFB-PET.

## Methods

### Patients

A consecutive number of 25 [^18^F]TFB-PET acquisitions combined with either CT or MRI of patients suffering from DTC (9 males and 16 females, median age 57) were included in this retrospective study (inclusion period: July 2018 to June 2019). Patients were referred to [^18^F]TFB-PET due to newly discovered increasing levels of thyroglobulin (TG), autoantibodies against thyroglobulin (antiTG), suspicious cervical lymph nodes, or combinations of the latter. Decision for [^18^F]TFB-PET examination was done on a case-by-case basis due to aforementioned clinical indication. Detailed patient characteristics are given by Table 1. The median time from thyroidectomy to [^18^F]TFB-PET was 5.1 [6.3] years. The median cumulated therapeutic dosage of [^131^I]iodine that was employed in the cohort until [^18^F]TFB-PET was 10 [10.4] GBq. All patients were treated with Levothyroxine to suppress thyroid-stimulating hormone (TSH) levels [2].

**Table 1:** Patient characteristics

Female/male	16 / 9
Median age in years [SD]	57.0 [16.3]
Median age at diagnosis in years [SD]	45.0 [15.0]
Papillary thyroid cancer	17 (68%)
Follicular variant	2 (8%)
Tall-cell variant	1 (4%)
Follicular thyroid cancer	8 (32%)
Minimal invasive	1 (4%)
Struma ovarii	1 (4%)
UICC TNM, 8th ed. 2017	
pT1	6 (24%)
pT2	4 (16%)
pT3	11 (44%)
pT4	2 (8%)
pTx	2 (8%)
pN0	5 (2%)
pN1	16 (64%)
pNx	4 (16%)
cM0	11 (44%)
cM1	14 (56%)
PUL	12 (48%)
OSS	2 (8%)
HEP	1 (4%)
Median TG, if measurable (ng/ml)	1.9 [1116.3]
Median antiTG, if measurable (IU/ml)	144.4 [1169.9]

[], standard deviation; (), percentage of all patients; PUL, lung metastases; OSS, bone metastases; HEP, liver metastases; TSH, thyroid-stimulating hormone; TG, thyroglobulin; antiTG, autoantibodies against thyroglobulin

### [^18^F]TFB preparation

[^18^F]TFB was prepared according to a literature procedure described previously by isotopic exchange of BF_4_^−^ with [^18^F]fluoride in hot hydrochloric acid and purified using an alumina column [16]. The product activity ranged between 6 and 8 GBq/batch. Radiochemical purities were determined by TLC and HPLC. The HPLC system was composed of a S1122 pump (Sykam, Fürstenfeldbruck, Germany); a S-2500 UV detector (KNAUER, Berlin, Germany); a GabiStar γ-detector (Elysia-Raytest GmbH, Straubenhardt, Germany); an Exsil ODS column, 5 μm, 250 × 4.0 mm (SGE Europe Ltd., Bucks, UK) with a mobile phase of 1-mM tetrabutylammonium hydroxide and 1.3 mM potassium hydrogen phthalate in water (pH 7.5) flowing at 1.0 ml/min, detection at λ = 254 nm; and the GINA Star software (Elysia-Raytest GmbH, Straubenhardt, Germany) ([^18^F]fluoride, t_R_ = 4.7 min; [^18^F]TFB, t_R_ = 11.2 min). Thin-layer chromatography (TLC) was performed on alumina TLC strips (Polygram Alox N/UV_254_, 40 × 80 mm, Machery-Nagel, Düren, Germany) with methanol as mobile phase. Strips were scanned using a radio TLC scanner (Elysia-Raytest GmbH, Straubenhardt, Germany). R_f_([^18^F]fluoride) = 0.0; R_f_([^18^F]TFB) = 0.8.

### [^18^F]TFB PET and [^131^I]iodine imaging

Thyrotropin alfa was administered 48 and 24 h before the administration of [^18^F]TFB and [^131^I]iodine. A median activity of 317.0 [63.0] MBq [^18^F]TFB was administered intravenously. The PET acquisition was initiated 40 min after tracer injection (Biograph mCT flow or Biograph mMR, Siemens Healthineers, Erlangen, Germany; acquisition speed: 2 min/bed position or 1.1 mm/s). Low-dose CT or MRI was acquired for attenuation correction of PET data. A median activity of 315.0 [516.8] MBq [^131^I]iodine was administered immediately after completion of the PET acquisition (a month delayed in one patient; only patient #12 received a therapeutic dosage of 3 GBq [^131^I]iodine). WBS and SPECT-CT acquisitions were done in accordance with guidelines (Discovery NM/CT 670 Pro, GE Healthcare, Chalfont St Giles, GB or Symbia T2, Siemens Healthineers, Erlangen, Germany) [17].

### Biochemical analysis

Blood levels of TSH, free triiodothyronine (fT3), free thyroxine (fT4), TG, and antiTG were measured on the day of the first thyrotropin alfa administration (TG/TSH, Elecsys assays and cobas e 801, Roche Diagnostics, Rotkreuz, CH; TG, assay and KRYPTOR hTg sensitive, BRAHMS GmbH/Thermo Fisher Scientific, Hennigsdorf, Germany). Stimulated TG levels were measured before [^131^I]iodine WBS acquisition.

### Statistical analysis

SPSS statistics 24 (IBM, NY, USA) was used for descriptive metrics and statistical testing. The McNemar and Wilcoxon method were used to test for statistically significant differences with the “exact” option of SPSS. H_0_ was rejected if *p* < 0.05. Values are presented as median with standard deviation (SD) in squared brackets.

## Results

A total number of 25 patients underwent [^18^F]TFB-PET, combined with either CT (*n* = 24) or MRI (*n* = 1; due to logistic reasons). In the following, PET-CT is used as phrase to refer to both PET-CT and PET-MRI acquisitions. Detailed patient characteristics are given by Tables 1 and 2. Patients were referred to imaging due to elevated TG levels (*n* = 19), elevated antiTG levels (*n* = 7), sonographically suspicious cervical lymph nodes (*n* = 10), or combinations of these findings. Therefore, all enrolled patients were regarded as suffering from recurrent DTC. Correlative [^131^I]iodine imaging was present for 25 patients. The median interval between [^18^F]TFB-PET-CT and [^131^I]iodine dxWBS/SPECT-CT was 3.0 [7.3] days (range: 2–39 days). Correlative [^18^F]FDG-PET-CTs were present for 21 patients. The median interval between [^18^F]TFB-PET-CT and [^18^F]FDG-PET-CT was 6.9 [10.8] months. No local therapy was performed between [^18^F]TFB-PET-CT and [^18^F]FDG-PET-CT.

**Table 2:** Detailed report of findings

ID	Finding	antiTG	[^18^F] TFB	[^131^I]iodine dxWBS/ SPECT-CT	[^18^F] FDG	CT	Histological verification
1	n/a	N	N	N	n/a	N	N
2*	Local recurrence	P	P	N	N	(P)	P
3	Multiple lung metastases	N	N	N	P	P	N
4	Cervical lymph node metastasis	P	P	N	P	N	N
5	Cervical lymph node metastasis	P	P	N	P	N	N
6	Lung metastasis	N	N	N	N	P	N
7	Lung metastasis	P	P	N	N	P	N
8	n/a	N	N	N	n/a	N	N
9	n/a	N	N	N	N	N	N
10	Cervical lymph node metastasis	N	P	N	P	N	P
11	n/a	N	N	N	N	N	N
12*	Bone metastases and local recurrence	P	P	P	N	N	N
13	Lymph node metastasis	N	P	P	N	N	N
14	Lymph node metastasis	N	N	N	P	N	N
15	Lymph node metastasis and local recurrence	N	N	N	P	N	N
16	n/a	N	N	N	n/a	N	N
17	Lung metastasis	N	N	N	n/a	P	N
18	Lung metastasis	P	N	N	N	P	N
19	Mediastinal lymph node metastasis	N	P	N	P	N	N
20	n/a	N	N	N	N	N	N
21	Local recurrence	N	P	N	P	N	N
22	Cervical lymph node metastases	N	P	N	P	N	N
23*	Lung metastasis	N	P	P	N	N	N
24	Lymph node metastasis	N	P	N	P	N	P
25*	Local recurrence	N	P	N	N	N	P

*Patient shown in figure

P, positive finding, i.e., suspicious for malignancy; N, negative finding; n/a, not available; DTC, differentiated thyroid cancer; TFB, tetrafluoroborate; FDG, fluorodeoxyglucose; CT, computed tomography; dxWBS, diagnostic whole-body scintigraphy; antiTG, autoantibodies against thyroglobulin (> 15 IU/ml = P; otherwise = N)

### [18F]TFB detection rate and reference standards

**Fig. 1:**
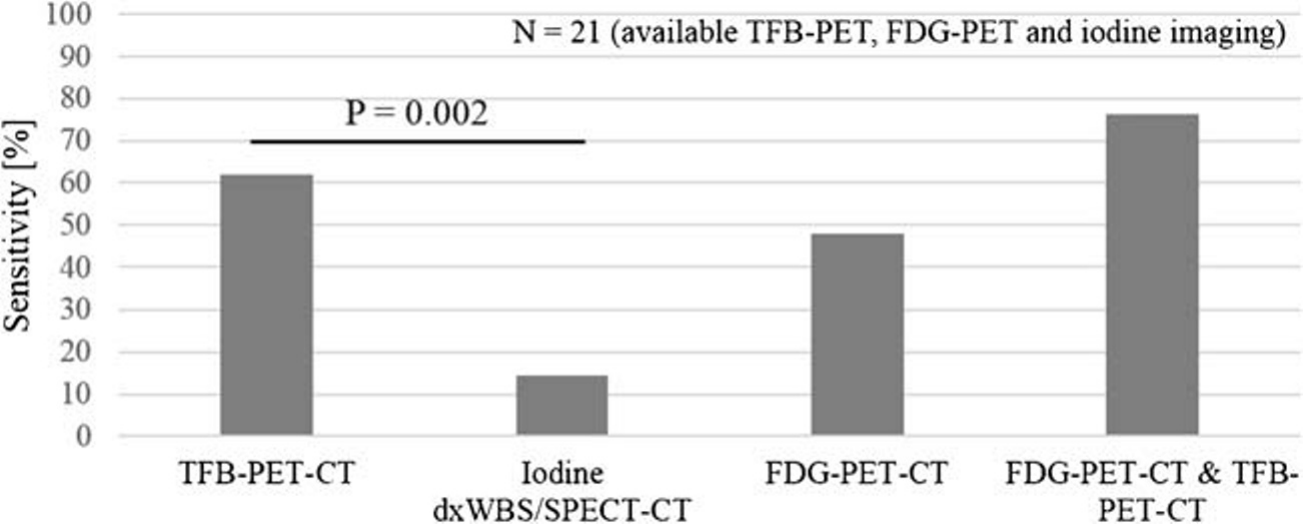
Sensitivity. Sensitivity of [^18^F]TFB-PET-CT and other imaging modalities for detecting recurrent DTC. Only patients with available [^18^F]TFB-PET-CT, [^18^F]FDG-PET-CT, and [^131^I]iodine dxWBS/SPECT-CT were regarded in this figure (*n* = 21)

The detection rate of [^18^F]TFB-PET-CT was 52.0% (*n* = 13). Details are given in Fig. 1. A total number of 27 lesions were detected by [^18^F]TFB-PET-CT. All lesions were delineated due to increased [^18^F]TFB-PET accumulation. Metastases or local recurrence were discovered in 10 additional patients compared to [^131^I]iodine dxWBS/SPECT-CT (Fig. 3). The detection rate of [^18^F]TFB-PET-CT was significantly higher compared to [^131^I]iodine dxWBS/SPECT-CT (recurrence detected in 13 vs. 3 patients; *p* = 0.002). A detailed list of findings is given in Table 2 (see also Figs. 4 and 5). Reference standard for [^18^F]TFB-PET-CT-positive patients (*n* = 13) was surgery together with histological examination (in 4 patients), a corresponding [^131^I]iodine accumulation (in 3 patients), [^18^F]FDG accumulation (in 5 patients), or morphological finding on CT (in 1 patient; lung metastasis). Therefore, the sensitivity of [^18^F]TFB-PET-CT was 52% (accuracy: 52%). The median [^18^F]TFB uptake of delineated lesions was 4.2 SUV_max_ [8.4].

Looking at patients who underwent both [^18^F]TFB-PET-CT and [^18^F]FDG-PET-CT, the detection rate of [^18^F]TFB-PET-CT was higher (51.9%) compared to [^18^F]FDG-PET-CT (47.6%), yet this difference was not statistically significant. In three patients, [^18^F]FDG-PET-CT discovered metabolically active metastases (median SUV_max_: 11.9) with no increased [^18^F]TFB accumulation. In contrast, [^18^F]TFB-PET-CT discovered lesions in six patients (local recurrence, lymph node or lung metastases), with no [^18^F]FDG accumulation. Combining [^18^F]FDG-PET-CT and [^18^F]TFB-PET-CT for staging of suspected recurrent DTC resulted in the highest sensitivity (64%; positive predictive value, 100%; accuracy, 64%).

In 6 patients, cervical lymph node metastases were suspected due to moderately increased [^18^F]FDG uptake (median SUV_max_: 4.5). Likewise, [^18^F]TFB-PET-CT revealed suspicious uptake of theses lymph nodes (median SUV_max_: 4.2). [^18^F]TFB-PET-CT-guided surgery together with histological analysis was performed in two patients. In both cases, lymph node metastases could be confirmed.

### [^131^I]iodine-SPECT-CT, [^18^F]FDG-PET-CT, and CT detection rates

**Fig. 2:**
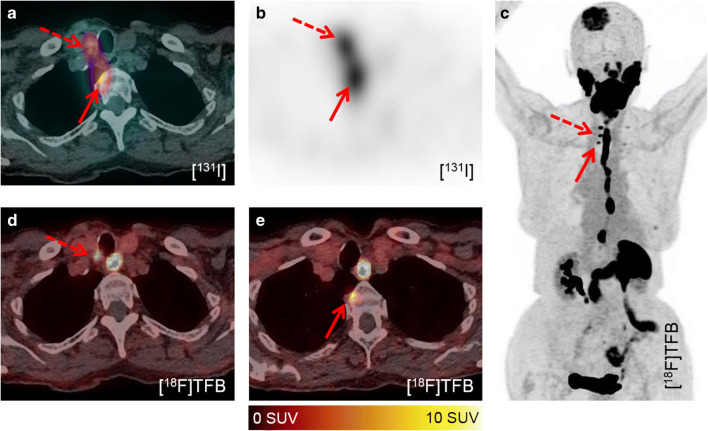
Superior image quality of [^18^F]TFB-PET-CT. In contrast to all other cases, patient #12 (92 years; TG, 5009 ng/ml; antiTG, 22.6 IU/ml; follicular DTC) received a therapeutic activity of [^131^I]iodine for imaging and therapy due to already known metastases. However, the delineation of local recurrence (dashed arrow) and a bone metastasis (arrow) is difficult in the SPECT-CT fusion (**a**). The transaxial SPECT reconstruction enables a better discrimination (**b**). However, [^18^F]TFB-PET-CT enabled superior delineation of metastases and local recurrence (**c–e**). Note additional bone metastases, e.g., skull

In three patients, [^131^I]iodine accumulating metastases were discovered by dxWBS and SPECT-CT (12.0%, one patient received a WBS with therapeutic dosage). In total, 11 lesions were detected by [^131^I]iodine imaging, which were significantly fewer compared to [^18^F]TFB-PET-CT (*p* = 0.001). All lesions were also detected by [^18^F]TFB-PET-CT, which additionally provided superior image quality (Fig. 2). Given histological CT or FDG-PET findings as reference standard, the sensitivity of iodine imaging was 12% (positive predictive value, 100%; accuracy, 12%). [^18^F]FDG accumulating local recurrence or metastases were delineated in 10 patients. The median uptake of these lesions was 5.2 SUV_max_. Morphological findings without [^18^F]TFB, [^18^F]FDG, or [^131^I]iodine uptake were present in patients with lung metastases only (*n* = 3, 12%).

### TG, TG-DT, and antiTG levels

Median TSH suppressed TG and antiTG levels for patients with [^18^F]TFB-positive findings were 2.46 [1566.9] ng/ml and 98.7 [144.7] IU/ml, respectively. For comparison, [^18^F]TFB-negative patients had median TG levels of 1.87 [4.4] ng/ml. Only one [^18^F]TFB-negative patient had elevated antiTG (3267 IU/ml). In six patients, no morphological or molecular findings were detected. Median TG level of these patients was 0.83 [1.2] ng/ml (no patient with elevated antiTG levels).

## Discussion

The performance of [^18^F]TFB-PET-CT in the diagnostic workup of patients with suspected recurrence of DTC was evaluated in the present study. To this end, 25 patients with sonographically suspicious cervical lymph nodes or elevated blood tumor markers (TG or antiTG) were included in this retrospective analysis. [^18^F]TFB-PET-CT detected local recurrence or metastases of DTC in significantly more patients compared to [^131^I]iodine dxWBS/SPECT-CT. Moreover, the accuracy of [^18^F]TFB-PET-CT was greater compared to [^131^I]iodine dxWBS/SPECT-CT.

It was shown previously that [^131^I]iodine WBS/SPECT-CT is inferior to TG levels in detecting patients that develop metastases or local recurrence [18]. Gonzalez Carvalho et al. stated that up to 25% of patients with recurrent DTC had two subsequent negative [^131^I]iodine dxWBS image acquisitions [18]. This indicates that [^131^I]iodine dxWBS is not ideal for detecting recurrent DTC. However, evidence of recurrent DTC by elevated TG levels does often not enable a localized therapy. Therefore, molecular imaging strategies are needed to localize recurrent DTC more sensitively than [^131^I]iodine dxWBS.

It has been shown previously that [^124^I]iodine-PET is superior to [^131^I]iodine dxWBS in detecting recurrent DTC [4]. However, [^124^I]iodine is not available in many departments of nuclear medicine [2]. Moreover, PET imaging with [^124^I]iodine requires a tracer administration days before image acquisition, which causes patient management issues. Finally, the image quality of [^124^I]iodine-PET is not preferable due to low signal-to-noise ratios. In contrast, [^18^F]TFB-PET-CT can easily be produced by a cyclotron equipped radiochemistry department and delivers excellent image to noise ratios and the image acquisition can be initiated minutes after tracer administration. Interestingly, patients 1 and 10 showed similar blood parameters (TG 0.55 ng/ml, antiTG < 15); however, only patient 10 showed suspicious [^18^F]TFB accumulation, which was histologically confirmed to be a metastasis. Therefore, [^18^F]TFB-PET-CT might be advisable in patients with minimally elevated tumor marker levels.

[^18^F]FDG-PET-CT imaging in DTC has been proposed to overcome the limitations of diagnostic [^131^I]iodine WBS [3, 10, 19–21]. A large multicenter study could demonstrate that [^18^F]FDG-PET has a higher sensitivity than [^131^I]iodine dxWBS [3]. Riemann et al. argued that this is partly caused by the tumor biology of DTC [3]: In case of recurrent DTC, a dedifferentiation of tumor cells might occur that decreases hNIS expression but increases [^18^F]FDG metabolism in turn [9]. This constellation is known as TENIS or flip-flop phenomena [3, 8, 9]. However, [^18^F]FDG positiveness together with negative [^131^I]iodine dxWBS does not prove complete dedifferentiation. It has been shown that [^124^I]iodine PET and therapeutic [^131^I]iodine WBS have a greater sensitivity than [^131^I]iodine dxWBS [4]. Additionally, [^18^F]FDG uptake of metastases might at least partly be caused by tumor infiltrating immune cells [22]. Thus, the rate of TENIS could be overestimated utilizing [^131^I]iodine dxWBS together with [^18^F]FDG-PET-CT. Therefore, [^18^F]FDG-PET and hNIS targeting molecular imaging are not equivalent but complementary.

The advantage of combining [^18^F]FDG-PET-CT with high-quality hNIS targeting molecular imaging is corroborated by the present study. Cervical lymph node metastases with moderate [^18^F]FDG accumulation but negative diagnostic [^131^I]iodine WBS/SPECT-CT were discovered in six patients. Thus, TENIS was suspected in these cases. However, [^18^F]TFB-PET-CT was positive thus suggested only partly dedifferentiation. Moreover, [^18^F]TFB-PET enabled a clear delineation of the metastases that lead to successful surgery in two cases, which enabled the histological confirmation of DTC. Two different patients of this study showed strong [^18^F]FDG accumulation of DTC metastases without evidence of functional relevant hNIS expression (negative [^131^I]iodine WBS and [^18^F]TFB-PET). Therefore, dedifferentiation and TENIS were assumed. Taken together, the combination of [^18^F]FDG-PET and [^18^F]TFB-PET seems reasonable due to their complementary nature. The combination of both imaging modalities achieved the highest sensitivity and accuracy (both: 62%) by sensitively detecting both differentiated and dedifferentiated recurrence of DTC.

The present retrospective study faces some limitations. First, the enrollment of patients was done retrospectively and is therefore prone to selection biases. For instance, many enrolled patients had only minimally elevated TG levels or had undergone [^131^I]iodine dxWBS/SPECT-CT imaging several times with inconclusive findings. Therefore, the pretest probability of positive imaging findings might be low. This could affect the transferability of the results to the clinical routine and underestimate the diagnostic performance of [^18^F]TFB-PET-CT. Moreover, only a relatively small patient collective was included due to the limited application of [^18^F]TFB-PET-CT in the clinical routine.

**Fig. 3:**
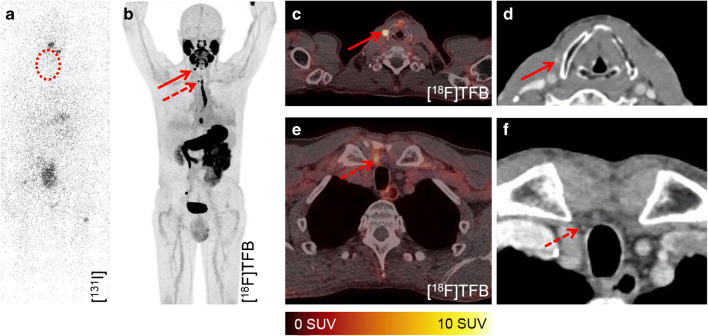
Detection of local recurrences and morphological correlates. No pathological accumulation of [^131^I]iodine is visible in the dxWBS (**a**), especially no cervical accumulation (dashed cycle). [^18^F]TFB-PET-CT enabled the detection of locally recurrent DTC at two sites (paralaryngeal, arrow, and retrosternal, dashed arrow (**b**, **c**, **e**)) in patient #2 (62 years; TG, 1.66 ng/ml; antiTG, 21.6 IU/ml; follicular DTC). Recurrence of DTC was histologically validated. Respectively, contrast enhanced foci were retrospectively detected in prior CT acquisitions (**c**, **e**)

Finally, [^18^F]TFB shows increased retention in the blood, which caused difficulties in the reading of head and neck acquisitions, especially when combined with low dose CT (note the blood pool in Fig. 3). Therefore, future studies should optimize the interval between injection of [^18^F]TFB and image acquisition. An increased interval between administration and image acquisition might result in lesser presence of [^18^F]TFB in the blood. Additionally, it might be warranted to combine [^18^F]TFB-PET with contrast enhanced morphology imaging to clearly delineate cervical blood vessels. This could facilitate the characterization of cervical [^18^F]TFB accumulating foci as either unspecific blood retention or cervical metastases.

**Fig. 4:**
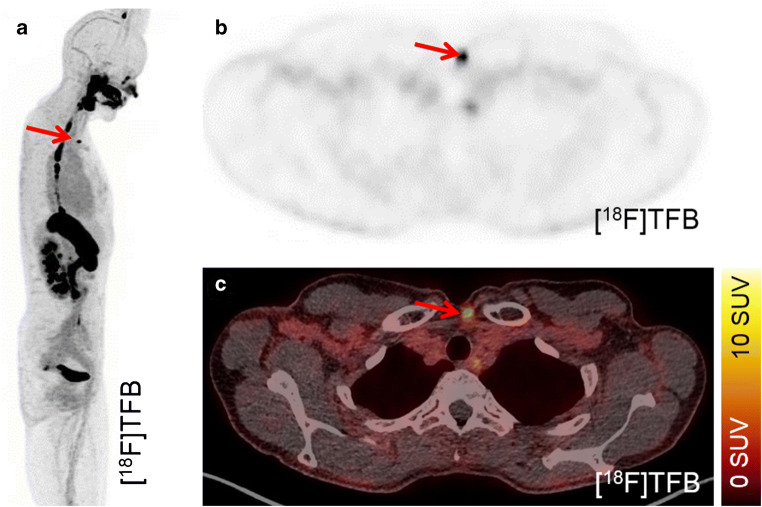
Detection of local recurrence. [^18^F]TFB-PET-CT enabled the clear delineation of local recurrence in patient #25 (arrow; 52 years; TG, 2.84 ng/ml; antiTG, < 15 IU/ml; follicular DTC). The recurrence of DTC was histologically validated

**Fig. 5:**
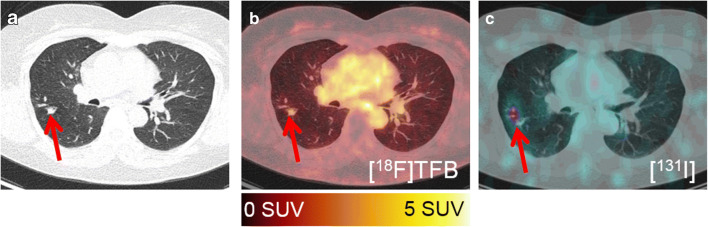
Detection of lung metastasis. A lung metastasis (arrow) of patient #23 (56 years; TG, 5.21 ng/ml; antiTG, < 15 IU/ml; papillary DTC) is detected by CT (**a**), [^18^F] TFB-PET-CT (**b**), and [^131^I]iodine SPECT-CT (**c**)

## Conclusion

[^18^F]TFB-PET-CT detected local recurrence or metastases of DTC in significantly more patients than [^131^I]iodine dxWBS/SPECT-CT. The combination of [^18^F]FDG and [^18^F]TFB PET-CT imaging achieved the highest diagnostic performance. Future studies evaluating [^18^F]TFB-PET-CT in recurrent DTC seem warranted.
